# Effectiveness of hands-on tutoring and guided self-directed learning versus self-directed learning alone to educate critical care fellows on mechanical ventilation – a pilot project

**DOI:** 10.3402/meo.v21.32727

**Published:** 2016-09-29

**Authors:** Kannan Ramar, Alice Gallo De Moraes, Bernardo Selim, Steven Holets, Richard Oeckler

**Affiliations:** 1 Division of Pulmonary and Critical Care Medicine, Mayo Clinic, Rochester, MN, USA; 2 Department of Anesthesiology, Mayo Clinic, Rochester, MN, USA

**Keywords:** fellows, critical care, pulmonary and critical care, education, mechanical ventilators

## Abstract

**Background:**

Physicians require extensive training to achieve proficiency in mechanical ventilator (MV) management of the critically ill patients. Guided self-directed learning (GSDL) is usually the method used to learn. However, it is unclear if this is the most proficient approach to teaching mechanical ventilation to critical care fellows. We, therefore, investigated whether critical care fellows achieve higher scores on standardized testing and report higher satisfaction after participating in a hands-on tutorial combined with GSDL compared to self-directed learning alone.

**Methods:**

First-year Pulmonary and Critical Care Medicine (PCCM) fellows (*n*=6) and Critical Care Internal Medicine (CCIM) (*n*=8) fellows participated. Satisfaction was assessed using the Likert scale. MV knowledge assessment was performed by administering a standardized 25-question multiple choice pre- and posttest. For 2 weeks the CCIM fellows were exposed to GSDL, while the PCCM fellows received hands-on tutoring combined with GSDL.

**Results:**

Ninety-three percentage (6 PCCM and 7 CCIM fellows, total of 13 fellows) completed all evaluations and were included in the final analysis. CCIM and PCCM fellows scored similarly in the pretest (64% vs. 52%, *p*=0.13). Following interventions, the posttest scores increased in both groups. However, no significant difference was observed based on the interventions (74% vs. 77%, *p*=0.39). The absolute improvement with the hands-on-tutoring and GSDL group was higher than GSDL alone (25% vs. 10%, *p*=0.07). Improved satisfaction scores were noted with hands-on tutoring.

**Conclusions:**

Hands-on tutoring combined with GSDL and GSDL alone were both associated with an improvement in posttest scores. Absolute improvement in test and satisfaction scores both trended higher in the hands-on tutorial group combined with GSDL group.

Physicians undergo exhaustive training to develop adequate skills for operation of invasive and non-invasive mechanical ventilators (MV) to treat complex critically ill patients in the intensive care units (ICU). Andragogy (including self-directed learning) is defined as ‘the art and science of helping adults learn’, and is based on five assumptions (1, 2). The assumptions are (1) adult learners are independent and self-directed, (2) they have accumulated a wealth of experience to use as a resource, (3) they integrate learning into the demands of daily ritual, (4) they desire a problem solving–based approach, and (5) they are driven by internal rather than external motivation (1, 2). Assumptions 1, 3, and 5 may very well apply to medical fellows interested in real-time problem solving while accumulating experience in MV, suggesting self-directed learning as a feasible educational delivery method to educate fellows as long as they are provided the appropriate resources. However, fellows may not have enough accumulated wealth of experience to use MV (assumption #2) to get the appropriate education on MV (1, 2).

Hands-on tutoring with an associated workshop is an educational method for fellows. While these workshops may be resource intensive to develop and maintain, they offer a dynamic, versatile, and interactive setting that may be more effective for training on MV. Also hands-on training and clinical simulations are helpful to engage the learners and improve the ability to retain information (3, 4). We are unaware of any study that has assessed the effectiveness of either of these two methods to educate fellows on MV. We hypothesized that fellows will achieve higher scores on standardized MV testing and greater satisfaction after participating in a hands-on tutorial workshop combined with guided self-directed learning (GSDL), compared to GSDL strategy alone.

## Methods

First-year Pulmonary and Critical Care Medicine (PCCM) fellows (*n*=6) and first-year Critical Care Internal Medicine (CCIM) (*n*=8) fellows were recruited. Baseline assessments of knowledge in invasive and non-invasive MV were performed using a pretest with 25 standardized multiple choice questions to all participants. The course faculty developed and validated the multiple choice questions. Each question was reviewed in detail by each faculty independently and their responses were used to make modifications to the questions. The educational methods were administered at the beginning of training, when both programs provide a similar curriculum, and fellows in either the CCIM or the PCCM program are at a similar level of knowledge and experience as related to MV.

The CCIM fellows were exposed to a GSDL on MV over 2 weeks. The GSDL resources consisted of carefully selected review articles, papers, didactic materials, and slides prepared by the MV workshop instructors, as well as access to video links explaining the various modes of MV and waveforms. The CCIM fellows were given sufficient time to review these materials at their own leisure after hours or during protected conference time over the 2-week period. The average time to review the resource materials was approximately 2 h. The PCCM fellows were exposed to the hands-on tutoring/workshop. As part of the workshop, the PCCM fellows had access to the same GSDL resources as the CCIM fellows, 1 week before the start of the workshop. The hands-on tutoring/workshop was conducted over two half days. The first half day was on non-invasive MV and consisted of a didactic lecture (for 1 h), that covered topics on continuous positive airway pressure (CPAP), bilevel positive airway pressure (BPAP), and other advanced modes such as adaptive servoventilation (ASV), and volume assured pressure support (VAPS). The lecture was followed by a hands-on tutorial and workshop using different case scenarios followed by debriefing sessions. The second half day was on invasive MV. A similar format was followed with a didactic lecture covering topics on basic respiratory mechanics and physiology, brief review of the pre-course materials, pressure–volume curve, and equation of motion. The lecture was followed by hands-on tutorial/workshop using the Puritan Bennett-840 ventilator and an iron lung as a simulator. Knobology followed by the basic modes of ventilation – volume and pressure control, and pressure support modes were covered using case scenarios.

The 25 multiple choice questions were readministered to each group following a 1-week washout period without access to educational materials. Blind scoring of the test sheets was done independently by the education program coordinator. Results were reported using mean and standard deviation (SD) and proportions. Matched pair analysis was conducted with the participants pretest considered as their own control. A post-intervention satisfaction survey was conducted for each group using a Likert scale question. This study was declared exempt by the Mayo Clinic Institutional Review Board

## Results

Out of a total of 14 fellows, 93% (6 PCCM and 7 CCIM fellows, a total of 13 fellows), completed all evaluations and were included in the final analysis. One CCIM fellow did not complete the posttest within the specified timeframe and was therefore excluded from the analysis. The CCIM and PCCM fellows scored similarly in the pretest (64% vs. 52%, respectively, *p*=0.13) (Table 1). Following their respective interventions, the posttest scores increased in both fellow groups; however, no significant difference was observed based on the intervention type (74% vs. 77%, *p*=0.39) (Table 1). The absolute improvement with the hands-on tutoring appeared to be slightly higher than the GSDL strategy, but did not reach statistical significance (change in improvement in score of 25% vs. 10%, *p*=0.07) (Table 1). Adjustments for prior length of ICU training among fellows did not affect the results. Improved satisfaction scores were overall significantly positive with the hands-on-tutoring/workshop group using the Likert scale (4.5 vs. 3.1, *p*=0.04).

**Table 1:** Results: standard testing score summary of CCIM and PCCM groups

	CCIM (*N*=7)		PCCM (*N*=6)		
			
Groups	Mean	SD (proportions)	Mean	SD (proportions)	p*
Pre-intervention	16.1 (64%)	2.4 (10%)	13.1 (52%)	2.4 (10%)	0.125
Post-intervention	18.5 (74%)	3.5 (14%)	19.3 (77%)	3.4 (14%)	0.388
Absolute improvement (Delta)	2.4 (10%)	2.5 (10%)	6.1 (25%)	2.6 (10%)	0.07
p**	0.01		0.03		

CCIM, Critical Care Internal Medicine; PCCM, Pulmonary and Critical Care Medicine.

* *p*-Value is done on individual analysis with non-parametric test (Wilcoxon signed-rank test).

** *p*-Value is with pair analysis of pre–post data where each volunteer is its own control (Wilcoxon signed-rank test).

## Discussion

Hands-on tutoring/workshop combined with GSDL and GSDL alone approaches were both effective with improvement in fellow posttest scores. However, the absolute improvement in test scores trended higher (not statistically significant), in the hands-on tutorial combined with GSDL group as did the satisfaction scores based on the Likert scale compared to the GSDL group alone.

Our study has limitations. The sample size was small, making statistical tests less reliable. Also, our results may not be generalizable to other institutions, as similar resources and time allotment for the instructors may or may not be available. Another potential limitation may be in the assignment of the intervention groups by training type. While this may seem to introduce bias in our sample, we know that both fellowships provide similar experiences and curriculum in the first 6 months of training in MV, and we accordingly selected first-year fellows who were at the start of training within each fellowship, thereby limiting the risk of bias. The traditional method of teaching at the bedside was also not taken into account as an isolated group in our study, and some of the first-year PCCM fellows had some rotations in the ICU prior to the hands on workshop. We used the same questions for the pre and posttest which creates a test–retest bias, and could have accounted for the improvement in fellow posttest scores. However, the washout period and not providing the answers immediately after the pretest may mitigate some of this bias.

Several factors contribute to the success or failure of a particular educational delivery method (5). These approaches do to not take into account the learner type, and it is likely that teaching complex tasks such as MV to fellows might need to incorporate variety of tools including classic lecturing, self-direct learning, and a practice component such as a hands-on tutorial/workshop or simulation. We need further studies to assess modern teaching methods.

## Conclusions

Hands-on tutoring combined with GSDL and GSDL alone were both associated with improvement in posttest scores. Absolute improvement in test scores trended higher in the hands-on tutorial group combined with the GSDL group versus the GSDL group, though satisfaction scores were higher in the combined group. These preliminary findings should be confirmed in a larger study.
